# Case studies of health economic analyses informing pharmaceutical health technology assessments for essential medicine selection and public-sector guidelines in South Africa

**DOI:** 10.1017/S0266462324000448

**Published:** 2024-12-12

**Authors:** Trudy D Leong, Jacqui Miot, Andy Parrish, Jane Riddin, Yasmina Johnson, Tamara Kredo

**Affiliations:** 1 Health Systems Research Unit, South African Medical Research Council, Cape Town, South Africa; 2 Health Economics and Epidemiology Research Office, Faculty of Health Sciences, University of Witwatersrand, Johannesburg, South Africa; 3 Walter Sisulu University, Mthatha, South Africa; Frere and Cecilia Makiwane Hospitals, East London, South Africa; 4 National Department of Health, Affordable Medicines Directorate, Essential Drugs Programme, Pretoria, South Africa; 5 Western Cape Government Health and Wellness, Western Cape, South Africa; 6 Division of Clinical Pharmacology, Department of Global Health and Division of Biostatistics and Epidemiology, Department of Medicine, Faculty of Medicine and Health Sciences, Stellenbosch University, Stellenbosch, South Africa

**Keywords:** universal health coverage, essential medicines, health technology assessment, economic analysis, clinical guidelines

## Abstract

**Background:**

Constrained resources under universal health coverage (UHC) necessitate a balance between medication costs and essential health system requirements. Policymakers practice priority-setting, as either implicit or explicit rationing, embedded in evidence-informed decision-making processes to guide funding decisions. Health technology assessment (HTA) is a method that may assist explicit evidence-informed priority setting. South Africa developed an official HTA methods guide in 2022, however before this, commissioning and performing economic evaluations was not standardized.

**Methods:**

We conducted a descriptive collective case study to explore the impact of economic analyses on the selection of, and access to, essential medicines in South Africa. Four cases were purposefully selected, and both official information and secondary data, including media reports, were reviewed. Data elements were extracted and organized in a matrix. Cases were reported narratively with a positivist epistemological approach, presenting the authors’ reflections.

**Results:**

We found economic analyses that reflected methodologies described in the HTA guide: international reference pricing, cost-minimization, cost-effectiveness, cost-utility, and budget impact analyses. Economic analyses informing the ‘resource-use’ domain in the GRADE evidence-to-decision framework supported decision-making, influenced market-shaping with price reductions of interventions through benchmarking (fosfomycin, flucytosine), improved equitable access nationally (flucytosine), and prioritized a defined patient group in a justifiable and transparent manner (bortezomib).

**Conclusion:**

A standardized HTA evaluation process guided by a nationally accepted framework is necessary for evidence-informed decision-making. Economic analyses (cost-effectiveness, affordability, and resource use) should be consistently included when making decisions on new interventions.

## Background

South Africa has one of the most inequitable health systems globally (1), where about 50 percent of the country’s health expenditure purchases health services for over 86 percent of the population in the public sector and the balance is spent on the private sector (2;3). Rational use of the public healthcare budget is imperative, and many countries have adopted the model World Health Organization (WHO) Essential Medicines List (EML) concept (4). The EML aimed to use a limited number of cost-effective essential medicines to improve healthcare outcomes and attain equitable and sustainable access to affordable medicines (5). The WHO EML may be adapted to country-specific priority needs and guides rational funding decisions to progress to universal health coverage (UHC) (6). Currently, in South Africa, the ministerially appointed non-statutory advisory National Essential Medicines List Committee (NEMLC) is responsible for maintaining the public-sector national EML and supporting standard treatment guidelines (STGs) through evidence-informed decision-making (7). South Africa has a two-tiered public and private health system and NEMLC decisions that inform public-sector medicine access also frequently inform Prescribed Minimum Benefits entitlements in the private sector (7). The Council for Medical Schemes, a statutory body established by the Medical Schemes Act (Act 131 of 1998) is responsible for developing the Prescribed Minimum Benefits, which are defined as a set of benefits that all private insurers are mandated to cover (7).

The South African NEMLC considers clinical, economic, and pragmatic aspects of medicine selection decisions, following the Grading of Recommendations Assessment, Development and Evaluation (GRADE) method (8;9). The WHO does not systematically analyze medicine costs during the selection process, and affordability in low-middle-income countries is a further selection consideration. The WHO EML has, until recently, consistently listed affordable products. However, Hwang et al. (6) and the NEMLC (10) have noted that since 2021 there has been inclusion of high-price medicines, nonstandardized across disease areas. It is, therefore, important that the cost-effectiveness and affordability of medicines should be assessed within the local context. HTA is defined as a multidisciplinary process that uses explicit methods to determine the value of health technology at different points in its lifecycle. The purpose is to inform decision-making promoting an equitable, efficient, and high-quality health system’ (11).

Since the inception of the South African EML in 1996, economic evaluations have evolved from cost-minimization to more complex model-based cost-effectiveness with budget impact analyses to better inform decisions using the GRADE approach (12). Although a more consistent approach is now used by the NEMLC to decide which medicines require an economic evaluation, this remains an *ad hoc* process dependent on available resources to conduct evaluations. More recently, the National Health Insurance (NHI) Act has been officially approved (13) and the National Department of Health (NDoH) Health Technology Assessment (HTA) methods guide (8) was developed to progress the UHC agenda using a more structured and standardized approach to commission and conduct economic analyses.

We aimed to describe four types of economic analyses and explore how they influenced the selection and access to essential medicines for the South African public sector.

## Method

Objective: To describe four different economic analyses and their influence on decision-making for selecting essential medicines in the South African public sector.

We used a descriptive collective case study design. The case study method allows in-depth scrutiny of an issue, process, or event in its real-life context (14). Out of approximately seventy economic analyses conducted between 2012 and 2022, four cases were purposefully selected to interrogate the various types of economic analysis methodologies described in the HTA methods guide, 2022 (8), across different levels of care (primary, secondary, and tertiary levels). These four cases were identified by reviewing secondary data – published data, reports, and documents; news items identified from local and national websites; other published information; as well as engagement with experts (authors) working in medicine policy. The data was extracted and organized in a matrix (Table 1), and a narrative description of each case was presented.

**Table 1.** Data extraction matrix for four economic analysis case studies

Technology	Type of economic analysis/es	Data sources	Analyses output	Decision	Impact	Implementation
Fosfomycin	International reference pricing	Economic analysis report; NEMLC report; SA-NDoH public-sector tender prices	Fosfomycin price was generally more expensive than in other countries	Fosfomycin not be included on the national EML (until the price is affordable) but was included on the therapeutic interchange database to provide for stock-outs of the current standard of care.	An estimated reference price could be determined for tendering purposes.	NEMLC report was published on the SA-NDoH Knowledge Hub platform for transparency and awareness, and the information assisted the public-sector tender processes.
3HP	Cost minimization	NEMLC evidence review comparing TPT to 12H; NDoH public-sector tender prices; provisioned public-sector price of 3HP	3HP was more costly than current standard of care, 12H	3HP not be included on the EML, until the price is affordable.	An estimated reference price could be determined for tendering purposes.	NEMLC report was published on the SA-NDoH Knowledge Hub platform for transparency and awareness, and the information assisted the public-sector tender processes.
Flucytosine	Cost-effectiveness and price-threshold	NEMLC evidence review and economic analysis comparing 2-week course of amphotericin B/ fluconazole, 2-week course of amphotericin B/ flucytosine, 1-week course amphotericin B/ flucytosine, 2-week course of oral fluconazole/ flucytosine and 1-week course amphotericin B/ fluconazole; SA-NDoH public-sector tender prices; private-sector prices; media reports of *Medecins Sans Frontieres’* (Doctors without borders) advocacy and stance regarding the high price of flucytosine (Daily Maverick, 20 April 2022)	The economic analysis suggests that a 60% price reduction for flucytosine would be acceptable for its inclusion on the national EML for cryptococcal meningitis. Publication of the analysis and threshold price created awareness amongst advocacy groups and the market players. An expression of interest advertised in the next public sector pharmaceutical tender, using the threshold price would prompt access to more affordable flucytosine.	Flucytosine will not be included on the national EML, until the product is registered, and the price is affordable.	An estimated reference price could be determined for tendering purposes.	NEMLC review and costing analysis published on the SA-NDoH Knowledge Hub platform and in a peer-reviewed journal for transparency and awareness, and the information assisted the public-sector tender processes.
Bortezomib	Cost-utility and budget Impact	NEMLC evidence review comparing bortezomib to the standard of care at the time (thalidomide plus corticosteroids)	Consistent clinical benefit as part of induction regimen, this unmet need considered to be possibly cost-effective, however should be restricted to specific parameters and monitored.	Bortezomib to be included on the EML (special access), for a particular indication, induction therapy for transplant eligible multiple myeloma patients (≤ 65 years of age). Data collection on use to be monitored by Pharmaceutical and Therapeutic Committees.	Access to expanded care for patients with multiple myeloma	NEMLC review and costing analysis published on the SA-NDoH Knowledge Hub platform for transparency and awareness.

3HP: 3-month course of rifapentine with isoniazid, 12H: 12-month course of isoniazid, EML: essential medicines list, NEMLC: National Essential Medicines List Committee, SA-NDOH: South African National Department of Health, TPT: tuberculosis preventive therapy.

A positivist epistemological approach (14) associated with deductive logical reasoning was used and objective results were generated, including reflections of the authors on lessons and challenges emanating from the findings.

### Case studies

The four case studies included various approaches and economic analyses: (i) international reference pricing, (ii) cost-minimization, (iii) cost-effectiveness, (iv) cost-utility and respective budget impacts for case studies three and four. We also described the impact of each analysis on improving access to the medicines under review (see Table 2). The information for case studies one (15), two (16), and four (17) was sourced from NEMLC reports accessible on an online portal that hosts products developed by the South African NDoH (https://knowledgehub.health.gov.za/). Case three was extracted from a 2021 peer-reviewed journal publication (18) and media reports (19;20).

**Table 2.** Summary of the key findings and impact of the four economic analyses on the selection and access of South African essential medicines

Case outline	Economic analysis	Key findings and implications
Economic analysis of fosfomycin for urinary tract infections in pregnant women	International benchmarking price analysis comparing global prices to SA price.	Comparable price analysis showed that SA price was considerably higher than global prices – the report was published, accordingly. Market shaping ensured more affordable products – by providing an expression of interest in fosfomycin to market players. Regular monitoring of prices is needed, as concern remains that once a medicine is recommended in the public-sector (with inclusion on the EML) the price may rise again.
Economic analysis comparing a 3-month course of rifapentine, and isoniazid therapy (3HP) compared to 12-month course of isoniazid (12H) for tuberculosis preventive therapy amongst people living with HIV (PLHIV) on initiation of antiretroviral therapy	Cost-minimization analysis comparing the local price of 12H to 3HP.	Study design should be considered when deciding to include or exclude completion rates in the economic analysis. Cost minimization analysis demonstrated that 3HP was more expensive than the current standard of care (12H). Availability of a registered fixed-dose 3HP tablet at a comparable or lower price to 12H may facilitate accessibility of 3HP going forward.
Economic analysis comparing a 2-week course of amphotericin B/ fluconazole, a 2-week course of amphotericin B/ flucytosine, a 1-week course amphotericin B/ flucytosine, a 2-week course of oral fluconazole/ flucytosine and a 1-week course amphotericin B/ fluconazole for the treatment of cryptococcal meningitis in PLHIV.	Cost-effectiveness and price-threshold analysis	The economic analysis suggests that a 60 percent price reduction of the price of flucytosine would be acceptable for inclusion on the national EML for cryptococcal meningitis. Publication of the analysis and threshold price created awareness amongst advocacy groups and the market players. An expression of interest advertised in the next public-sector pharmaceutical tender, using the threshold price would prompt access to more affordable flucytosine.
Economic analysis comparing bortezomib for induction treatment prior to autologous stem cell transplant in multiple myeloma patients compared to standard chemotherapies	Scoping of published economic evaluations and Health Technology Assessments. Cost-utility with budget impact analysis.	The addition of bortezomib to the induction regimen addresses an unmet clinical need in multiple myeloma patients eligible for autologous stem cell transplantation. Prioritization of bortezomib for use by a defined patient group was conducted justifiably and transparently.

3HP: 3-month course of rifapentine with isoniazid, 12H: 12-month course of isoniazid, EML: essential medicines list, NEMLC: National Essential Medicines List Committee, SA-NDOH: South African National Department of Health, TPT: tuberculosis preventive therapy.

#### 1) International reference pricing (conducted in 2016) to shape the market

*The case*: Local price of a single dose of fosfomycin compared to other international prices in 2016 including low-middle income countries.

*Context:* The STG and EML for primary care recommended ciprofloxacin (12-hourly for 7 days) for adult urinary tract infections (UTIs), in 2012. As quinolones are contra-indicated in pregnancy, oral amoxicillin/clavulanic acid (administered 12-hourly) was preferred in that situation, and the STGs and EML were updated in 2014. In 2016 local surveillance revealed a favorable sensitivity profile for single-dose oral fosfomycin, which at the time of that analysis was only available in the private sector, and three to four times more expensive than a course of amoxicillin/clavulanic acid.

*Objective*: To investigate comparative global prices of oral fosfomycin for the treatment of community-acquired UTI in pregnant women with local prices.

*Economic analysis:* International benchmarking price analysis of ex-manufacturer pricing at a set point in time, using an average exchange rate for a defined period to convert to South African currency (ZAR) (21). Details of the comparative global price analysis are available at: knowledge.hub.co.za.

*Data collection*: Prices were sourced locally from private-sector, public-sector, and publicly accessible databases internationally – as stated in the published NEMLC report (15).

*Key findings:* A 2016 analysis demonstrated that compared to other countries, South Africa’s access price (private-sector) for oral fosfomycin 3 grams (ZAR149.63, USD10.18) was the highest ex-manufacturer price in the international private sector, while published international prices for the public-sector use were considerably lower – see Table 3 and the published NEMLC report (15). Due to its high cost, fosfomycin was not included in the EML, with a planned review if the price fell. Publication of the NEMLC report that described the rationale for the NEMLC decision, and inclusion of fosfomycin in the published therapeutic interchange database and in a public-sector tender with a reference price, created market awareness of the South African NDoH’s intent. The access price for the public sector per unit was subsequently reduced to ZAR94.06 (USD7.07) (37 percent less than the 2016 South African private-sector price). The NEMLC decision was subsequently reviewed, and fosfomycin was included in the national EML. Thus, market-shaping to ensure more affordable products was enacted and an expression of interest in fosfomycin to market players was advertised through the public-sector tender process. As the analysis was conducted in 2016, the current public-sector contract price of fosfomycin 3 grams (single sachet) was sourced for March 2024 as ZAR42.00 (USD2.23) per unit (22), which was considerably lower than the price of the cheapest generic in the private sector of ZAR182.19 (USD9.69), possibly due to scale of volume (23).

**Table 3.** Comparison of 2016 prices for fosfomycin from various countries

Country	Trade name	Manufacturer price in South African rands in the private-sector, 2016 (USD)	Manufacturer price in South African rands in the public-sector, 2016 (USD)
South Africa	Urizone®	ZAR149.63 (USD10.18)	-
Austria	Monuril 3 g granulate®	-	ZAR57.09 (USD3.88)
Hungary	Monural®	ZAR36.58 (USD2.49)	ZAR36.58 (USD2.49)
	Fosfomycin Exeltis®	ZAR21.93 (USD1.49)	ZAR21.93 (USD1.49)
Turkey	Monurol®	-	ZAR49.04 (USD3.33)
Czech Republic	Rapidnorm 3000 mg®	ZAR82.82 (USD5.63)	-
Russia	Monural®	ZAR83.27 (USD5.66)	-
Canada	Monural®	ZAR78.85 (USD5.36)	-

g: gram, mg: milligram, USD: United States Dollar, ZAR: South African Rand.

*Implications:* Despite the reduced price that was subsequently offered for public-sector use, there is a possibility that market players might increase the price of fosfomycin once the medicine is listed on the national formulary. Thus, there is a need for continuous monitoring of the market to determine whether disinvestment of fosfomycin would be required. Consideration of benchmarking other antibiotics that could be used for UTI amongst pregnant women would have been beneficial for completeness of decision-making. Ongoing collaboration and consultation with end-users of policy recommendations regarding price and usage volumes is likely to be helpful.

*Comparison with the 2022 HTA methods guide:* This 2016 analysis also reviewed prices from other low-middle income countries and did not restrict the basket of countries to Australia, New Zealand, England, and Wales, which are high-income settings, as per the HTA methods guide. The rationale for this recommendation in the HTA guide merits review.

#### 2) Cost minimization analysis (conducted in 2016, updated in 2019) directed by primary evidence synthesis

*The case*: The local price of a treatment course of 12H (12 months of isoniazid) compared to the public-sector price of a three-month course of fixed-dose combination rifapentine and isoniazid (3HP) for adults living with HIV (PLHIV) and initiated on antiretroviral therapy (ART).

*Context:* The high tuberculosis (TB) burden in South Africa (estimated prevalence of 852 cases per 100,000 population, 95 percent CI 679–1026), and more so amongs PLHIV (1,734 cases per 100,000 population, 95 percent CI 1,219–2,249) (24) warrants directed strategies to reduce spread of this disease. Increasing active case-finding, (25) effective work practices (particularly in health facilities), and other TB infection prevention and control measures are described in the literature (26). Global guidelines advocate for TB preventive therapy (TPT) to reduce progression to active disease (27), especially amongst PLHIV (28). Stakeholders asked whether the shorter course of 3HP would improve completion rates amongst PLHIV initiated on ART compared to the standard of care recommended in national guidelines (12H). A review of the evidence was conducted (29) and 3HP was shown to be noninferior to 9‐12H, with a slightly improved safety profile. Improved 3HP completion rates were already factored into the modified intent-to-treat analysis.

*Objective*: This case study investigated the option of 3HP compared to 12H for TPT amongt PLHIV on initiation of ART.

*Economic analysis*: Cost-minimization analysis.

*Data collection*: Prices were sourced locally from the public-sector and market intelligence provided the provisional 3HP public-sector price stated in the published NEMLC report (16).

*Key findings:* The cost of a course of 3HP, 900/900 milligrams (mg) weekly for 3 months, was calculated as ZAR350.84 (USD24.28), which was more costly than the estimated price of 12H (300 mg daily for 12 months) of ZAR211.44 (USD14.63) and of 9H (300 mg daily for 9 months) estimated at ZAR158.58 (USD10.97).

*Implications:* 3HP could be considered as an alternative to 12H as TPT for PLHIV initiated on ART, given the non‐inferiority in efficacy and slightly improved safety profile of 3HP, but price parity would be required. Thus, NEMLC recommended that 3HP not be included in the national EML until there is price parity, given the constrained health budget. Some stakeholders believed that improved completion rates for 3HP should have been considered in the economic analysis. Notably, for clinical effectiveness, observational data are generally not preferred over randomized controlled data in clinical decision-making, due to confounding (30). More recently, authors of a cost-effectiveness systematic review mentioned that ‘limited, existing evidence suggests 3HP may not be cost-effective in low-income and high-HIV-coinfection settings’ (31). The May 2023 contract price for twenty four tablets of rifapentine 150 mg, ZAR133.10 (USD7.32), was 18 percent higher than in 2019, while isoniazid 300 mg, twenty eight tablets of ZAR16.86 (USD0.93) had decreased by 5 percent (32). Rifapentine was listed as a non-EML item on the national pharmaceutical contract as it is more costly compared to the current standard of care and has not been included on the national EML for routine use at public-sector facilities. Availability of a registered fixed-dose 3HP tablet at a comparable or lower price to 12H may facilitate accessibility of 3HP going forward.

*Comparison with the 2022 HTA methods guide:* This case could be further strengthened by considering additional components as proposed by the HTA methods guide: identifying other direct costs such as drug administration, monitoring, and safety costs; conducting sensitivity analyses to test the robustness of any assumptions; and subgroup analysis as supported by relevant clinical evidence.

#### 3) Cost-effectiveness analysis with threshold price analysis (conducted 2018/2019) to guide procurement

*The case*: This case study investigated the cost-effectiveness of flucytosine in reducing mortality amongst PLHIV with cryptococcal meningitis, with an indication of the willingness-to-pay price and supporting estimated budget impact analysis.

*Context:* Cryptococcal meningitis (CM) mortality in PHLIV remains high despite current treatment options of amphotericin B and fluconazole. Flucytosine in combination with amphotericin B reduces mortality and toxicity (33) and is included in WHO guidelines (34). However, access to flucytosine remains uncertain and in many countries, the price is far higher than other treatment options (35).

*Objective*: To evaluate flucytosine and amphotericin B as initiation therapy for CM in PLHIV compared to the current standard of care (amphotericin B and fluconazole) in the South African public sector.

*Economic analyses*: Cost-effectiveness and price-threshold analyses comparing a two-week course of amphotericin B/fluconazole, two-week course of amphotericin B/flucytosine, one-week course of amphotericin B/flucytosine, two-week course of oral fluconazole/flucytosine, and one-week course amphotericin B/fluconazole (18).

*Data collection*: A decision tree analysis was conducted with a time horizon of up to 25 years and the perspective was that of the healthcare payer. An ingredients-based approach was used to calculate local healthcare costs, based on local prices and resource utilization data for the South African context.

*Key findings:* The lowest cost treatment was the one-week amphotericin B/fluconazole arm; however, this also had the poorest outcomes. The one-week amphotericin B/flucytosine course was the most cost-effective (USD119/QALY) and had an incremental per-patient cost of USD293 per year compared to the current standard of care. The model was sensitive to life expectancy and hospital costs, particularly infusion costs and length of stay, which are important indicators of possible further reduction in costs as flucytosine is administered as an oral treatment.

*Implications:* Based on the results of this economic analysis the NEMLC made the decision not to include flucytosine for CM on the national EML, pending a price reduction of 60 percent. In December 2021, flucytosine was registered with the South African Health Products Regulatory Authority and was subsequently listed on the pharmaceutical tender at the proposed reduced price of USD98.87 for a pack of 100 flucytosine 500 mg tablets (36). This will allow for equitable public-sector access nationwide to flucytosine, a critical medicine for the treatment of CM in HIV.

*Comparison with the 2022 HTA methods guide:* This analysis, conducted in 2018/9 was aligned with the HTA methods guide and the Consolidated Health Economic Evaluation Reporting Standards CHEERS statement (37). However, no explicit statement was mentioned in the flucytosine cost-effectiveness analysis (CEA) journal article (18) pertaining to the need for subgroup analysis and whether a scoping review was conducted.

#### 4) Cost-utility analysis (conducted 2020/2021) for an unmet clinical need

*The case*: Bortezomib has been shown to be effective for induction treatment prior to autologous stem cell transplant (ASCT) in multiple myeloma; however, it costs more than conventional chemotherapies.

*Objective*: This case study investigates the addition of bortezomib to the induction regimen for transplant-eligible multiple myeloma patients.

*Context:* A goal of frontline treatment of multiple myeloma is to maximize tumor reduction. Induction therapy before transplantation can influence post-transplant results. The effectiveness of bortezomib for induction treatment prior to ASCT in multiple myeloma patients has been demonstrated in several randomized, open-label phase III trials (38–40).

*Data collection*: Prices were sourced locally from public- and private-sector databases – as stated in the published NEMLC report (17).

*Economic analyses:* As part of initial scoping, a review of published economic evaluations and International Health Technology Assessments was undertaken. A decision tree model was used to determine the cost-utility of combination bortezomib, thalidomide, and dexamethasone compared to thalidomide and dexamethasone. A budget impact analysis was conducted to address affordability.

*Key findings:* The cost analysis showed the addition of bortezomib would result in both an incremental cost and incremental effect with an incremental cost-effectiveness ratio of ZAR33,784 (USD2,284) per QALY gained. This was deemed to be cost-effective in this setting. Approximately 200 transplant-eligible multiple myeloma patients in the public sector could be treated annually with bortezomib, with a calculated net budget impact of ZAR 1.37 million (USD 0.09 million) to ZAR 2.74 million (USD 0.19 million).

*Implications:* The cost analysis allowed for the inclusion of bortezomib on the EML as an adjunctive induction regimen for transplant-eligible multiple myeloma patients enabling this item to be procured on the national public-sector pharmaceutical tender, which would foster public-sector pricing equity across the country. Access to bortezomib addressed an unmet clinical need. Monitoring use and patient outcomes by provincial Pharmaceutical and Therapeutics Committees will ensure appropriate investment, but this may be a challenge for busy healthcare providers and complete information may not be forthcoming.

*Comparison with the 2022 HTA methods guide:* Components of the analytical framework were included in this case report – scoping of various sourced HTA reports, appraised for methodological and context applicability, and subgroup analyses were presented in the sensitivity analyses. However, in the final CEA report, discounting or the time horizon were not explicitly described.

### Lessons learnt – challenges and opportunities

Across the case studies, the clinical evidence synthesis of benefits and harms informed the type of economic analysis that followed. Where interventions were found to be cost-effective, budget impact analyses were conducted to assess affordability. Economic evaluations were done implicitly in these case studies; however, this level of cost analysis is not always necessary for all decisions. Most economic analyses that informed NEMLC recommendations were conducted prior to the development of the NDoH 2020–2027 HTA methods guide and contributed to its development. The 2019/2020 bortezomib economic analysis was guided by the HTA methods guide. The guide provides a systematic approach to economic evaluations that should be considered for the review of technologies for selection decisions. This framework also enables the standardization of processes. However, the guide should be reviewed regularly and strengthened in certain areas such as the basket of countries for comparative price analysis. The HTA methods guide outlines a systematic approach to selecting the most appropriate type of economic analysis to be conducted, based on the nature and context of the decision problem, the requirements of the decision-maker, the type of information that is required to inform the decision, and where the uncertainty lies. The guide also provides information on the resources and the level of effort required for each type of analysis.

Due to scarce in-house technical skills and limited funding, donor funding was used to obtain external support from academia and public health economistsin three of the four case studies. The undertaking of good quality HTAs could be facilitated by capacity development for institutional technical personnel, ministry technical teams, professional societies, and university departmental staff. International entities such as the National Institute of Health and Care Excellence (NICE), commission external academic units to conduct HTA, and a similar model may be feasible in South Africa. This would require efficient coordination and adequate and sustainable funding. There is also a possibility of adapting some economic analyses, often conducted in high-income settings, to the South African context – however open economic models may not be readily available. Furthermore, sourcing data and adapting models appropriately to the South African setting still requires significant resources and technical capacity.

Estimating a benchmark price for cost-prohibitive medicines creates market interest with the opportunity for improved security of supply and more affordable prices. For market awareness, the NEMLC recommendations regarding non-EML medicines are published in peer-reviewed journal articles, as technical reports on the NDoH Knowledge Hub platform, or advertised on public-sector pharmaceutical tenders with a reference price. Once an essential medicine is included on the national formulary at an affordable price, monitoring of the market price is required to ensure that the intervention does not become unaffordable over time, especially when price is the variable that informs the selection decision. If cost-prohibitive, the intervention may be disinvested and removed from the EML, and an alternative agent may be sought.

## Discussion

This review demonstrates that different economic evaluation methods (cost-minimization, cost-utility, and cost-effectiveness analyses) and pricing mechanisms (international reference pricing), influenced NEMLC decision-making and impacted selection and access to medicines. Value-based pricing was estimated using ICERs and willingness-to-pay thresholds derived from economic evaluations, whereas international reference pricing was used to negotiate drug prices (41).

Using the HTA methods guide, a framework is provided for standardizing processes for the various economic analyses that are most appropriate for the particular decision. The cases that were selected for this analysis were considered to be of good quality. They did not consistently follow the requirements described in the methods guide, but this would probably not have impacted the outcome of the evaluations, or the decisions taken. It is important, however, to have a robust process in place where the quality of the economic evaluation can be assessed on an international and standardized platform. More recently a Scoping for Economic Evaluations template is being piloted with the NEMLC to determine *a priori* whether and what type of evaluation is required and to ensure it answers the question in an appropriate and relevant way.

South Africa has a two-tiered inequitable health system (42) and there are severe healthcare budget constraints that necessitate economic evaluations for evidence-informed decision-making and rational resource use. Consideration of affordability for EML inclusions/exclusions is paramount. In the context of poor economic growth, and reduced tax revenues, the healthcare budget 2023/2024 has resulted in a severely resource-constrained health sector (43). Thus, explicit priority-setting mechanisms are vital for the efficient allocation of resources.

WHO’s EML and guideline processes both intend to increase access to essential medicines and care, although as a result of their different decision-making processes, recommendations may be divergent. In South Africa, the STGs guide the implementation of the national EML (44).

Dedicated funding is allocated annually to the pharmaceutical budget in each of the nine South African Provinces, but this, is in the absence of explicit priority setting and funding, is generally allocated in an *ad hoc* implicit manner. Going forward, the NHI Act, will provide for funding of packages of care (benefits) that will be selected through a priority-setting exercise.

The case studies showed that economic evaluations contributed to the overall decision of including or excluding listing on the South African EML, and when made public and transparent, stimulated the market in terms of access and price. Price negotiations are not actively practiced for essential medicines at the South African NDoH with consideration of the Public Finance Management Act (45) except in the context of advertised/awarded tenders where benchmarking with the provision of a “willingness-to-pay” price encourages submission of better prices. Publishing technical reports strengthens transparency and provides an opportunity for price negotiations with market players. Thus, availability of these technical reports on the NDoH Knowledge Hub and other platforms should be encouraged for wider dissemination of decision recommendations.

South Africa is committed to UHC and the NHI Act stipulates that HTA informs decision-making and the benefits package. However, the Act does not provide specific details and HTA is yet to be institutionalized. A landscape analysis in Southeast Asia reported that in Indonesia, the Philippines, and Vietnam, UHC and the supportive processes were nascent. However, four countries in the analysis (Brunei Darussalam, Malaysia, Singapore, and Thailand) provide UHC with an established healthcare-priority setting system utilizing HTA (46). Three countries were similar to South Africa, namely, Cambodia, Lao People’s Democratic Republic, and Myanmar, where priority setting and HTA are conducted on an *ad-hoc* basis (with consideration of the political agenda during priority-setting in some countries) (46). Despite the lack of nodal HTA agencies, there is a commitment to progress toward UHC. Systematic implementation of HTA requires legislation, technical capacity, strong coordination, adequate financial resources, accountability by all stakeholders (all levels of government, healthcare professionals and providers, patients, academia, public, and industry representatives), and raising awareness of HTA to minimize misinformation and facilitate UHC progression (46).

Limitations of this study include that only four case studies were reviewed, and a more comprehensive review of all economic analyses conducted for review by NEMLC may assist in monitoring and evaluating local HTA implementation. Medicine selection decisions are guided by the GRADE approach, which considers multiple criteria besides resource use – efficacy, safety, feasibility, acceptability, and equity. Thus, the costing analyses would be considered in the context of the additional factors that informed the medicine selection decision.

The positivist epistemological approach used in this collective case study research focuses on testing and refining theory based on findings, but does not consider the researcher’s role in influencing these findings (14).

## Conclusion

A standardized HTA evaluation process, underpinned by a nationally accepted legislated HTA framework is necessary for evidence-informed selection of and improved access to essential medicines for the South African public health sector. The legitimacy of decision-making lies in the transparency of steps taken to reach a recommendation, with efficacy, safety, feasibility, acceptability, equity as well as affordability all contributing to the final decision. The impact of these case studies was to improve equitable access to essential medicines using transparency, robust economic methodologies, and market-shaping.
